# CERENKOV3: Clustering and molecular network-derived features improve computational prediction of functional noncoding SNPs

**Published:** 2020

**Authors:** Yao Yao, Stephen A. Ramsey

**Affiliations:** 1. School of Electrical Engineering and Computer Science, Oregon State University; 2. Department of Biomedical Sciences, Oregon State University Corvallis, OR 97330, USA

**Keywords:** SNP, GWAS, noncoding, rSNP, clustering, molecular network, machine learning

## Abstract

Identification of causal noncoding single nucleotide polymorphisms (SNPs) is important for maximizing the knowledge dividend from human genome-wide association studies (GWAS). Recently, diverse machine learning-based methods have been used for functional SNP identification; however, this task remains a fundamental challenge in computational biology. We report CERENKOV3, a machine learning pipeline that leverages clustering-derived and molecular network-derived features to improve prediction accuracy of regulatory SNPs (rSNPs) in the context of post-GWAS analysis. The clustering-derived feature, locus size (number of SNPs in the locus), derives from our locus partitioning procedure and represents the sizes of clusters based on SNP locations. We generated two molecular network-derived features from representation learning on a network representing SNP-gene and gene-gene relations. Based on empirical studies using a ground-truth SNP dataset, CERENKOV3 significantly improves rSNP recognition performance in AUPRC, AUROC, and AVGRANK (a locus-wise rank-based measure of classification accuracy we previously proposed).

## 1. Introduction

### The rSNP detection problem

Genome-wide association studies (GWAS) are increasingly being used to map the genes that underlie human polygenic traits. GWAS have uncovered variant-to-trait associations in thousands of studies collectively involving millions of individuals.^1^ Functional interpretation of genetic loci identified through GWAS has primarily focused on *coding regions* in which SNPs can be explained based on amino acid changes;^2^ however, 90% of human GWAS-identified SNPs are located in *noncoding* regions^3^ from which it is difficult to pinpoint the regulatory SNP (or rSNP) that is causal for trait variation.^4^

With the tremendous increase in genomic and functional genomic datasets, computational data-driven approaches have become a mainstay of functional rSNP prioritization, although the rSNP identification problem remains fundamentally challenging. While some unsupervised approaches, which do not involve training based on an example set of experimentally validated rSNPs, have been proposed,^5–15^ evidence from our work^16,17^ and others’^18–21^ suggests that supervised approaches in general have superior rSNP detection accuracy. In addition, the growth of literature-curated databases of experimentally validated rSNPs^22–24^ has stimulated the development of supervised approaches. A variety of supervised classification algorithms have been proposed, including the SVM,^11,13,18,25^ naïve Bayes,^26^ ensemble decision tree algorithms,^19,20,27^ probabilistic graphical models,^12,28^ deep neural networks,^14,21,29^ weighted sum of feature ranks,^30^ and our work using regularized gradient boosted decision trees^16,17^ and deep residual networks.^31^ Recently, several hybrid methods have been proposed such as combining recurrent and convolutional neural networks^21^ and combining deep neural networks with regularized gradient boosted decision trees.^29^ In addition to binary classification approaches, regression-based approaches have been used for the rSNP detection problem.^32,33^

For rSNP detection, as with other machine-learning problems, the features (in this case, SNP features) are as important as the classification algorithm. Consequently, various types of SNP annotations that correlate with functional rSNPs have been used,^34^ for example, phylogenetic sequence conservation and expression quantitative trait locus (expression QTL, or eQTL) association^35^ scores. Furthermore, studies have shown that increasing the diversity of SNP annotation features improves rSNP detection, and thus there has been a steady increase in the number of features used in machine-learning approaches for this problem.^10,18–21,26,27,29^ In our previous work^17^ we reported a model (Computational Elucidation of the REgulatory NonKOding Variome, CERENKOV) with a 246-dimensional feature space that clearly outperformed some models^20,21,29^ with significantly higher-dimensional feature spaces. This suggested that feature correlation within, and sparsity of, high-dimensional feature space may weaken the improvement of rSNP detection accuracy. Therefore, how to identify and integrate various types of rSNP correlates remains a key challenge for accurate rSNP detection.

### Our previous CERENKOV methods

Our previous classifier, CERENKOV,^17^ had four key innovations. First, we selected a reference set of SNPs to represent noncoding loci that would be expected to be encountered in a post-GWAS analysis, based on population minor allele frequency.^17^ Second, we used a regularized gradient boosted decision tree (XGBoost) classification algorithm,^36^ which we found has superior rSNP recognition performance to Random Forest and Kernel SVM. Third, we engineered 246 SNP-level features from phylogenetic, genomic, epigenomic, chromatin structural, cistromic, population genetic, replication-timing, and functional genomic datasets. Fourth, CERENKOV incorporated a locus-wise rank-based measure of classification accuracy, AVGRANK,^17^ which more realistically models the costs associated with incorrect predictions in post-GWAS analysis than typical measures like area under the receiver operating characteristic curve (AUROC) or area under the precision-recall curve (AUPRC). We compared the accuracy of CERENKOV to nine previously published rSNP recognition models^17^ and found that CERENKOV’s performance significantly improved upon the nine other models, by AUPRC, AUROC, and AVGRANK.

More recently, we reported on CERENKOV2,^16^ which improved performance over CERENKOV by leveraging insights into the data-space geometry of the problem. In addition to using a 2.5-fold expanded reference set of SNPs (the OSU18 SNP set which has 39,083 SNPs for model benchmarking), we incorporated new features that are based on likelihood ratios of average SNP-to-neighboring-SNPs distances for various types of distance measures. By taking account geometric properties of the distribution of SNPs in data space, CERENKOV2 achieved significantly better rSNP recognition performance than CERENKOV and (as with CERENKOV) it outperformed the next-strongest rSNP detection tool, GWAVA.^19^

### CERENKOV3: new clustering-derived and network-derived features

#### Clustering:

Clustering is a widely used technique for statistical data analyses. In GWAS, SNP clustering can help detect groups of similar SNPs that are amenable to classification using group-wise models.^37^ To find useful SNP partitions, one option is to use specific domain knowledge to group the SNPs, for example by target genes and/or functional pathways. Another option is to use hierarchical clustering methods which rely on a distance measure between the SNPs. For example, SNPs can be clustered based on their pairwise relation given by stagewise regression coefficients using average linkage and the result of clustering helps alleviate the dimensionality problem when training deep Boltzmann machines.^38^ Therefore, we hypothesized that deriving a feature to explicitly account for SNP clustering could improve performance for rSNP detection in the context of supervised classification.

#### Molecular network:

Molecular networks, especially gene regulatory networks (GRN), are also key to the rSNP detection problem because molecular networks mediate the effects exerted by rSNPs on trait population variation. Therefore we hypothesized that mapping SNPs to a molecular network, and deriving features from the vantage points of SNPs in the network, would benefit rSNP detection. Construction of such a network is greatly aided by the recent availability of datasets from studies of direct DNA contacts utilizing assays such as Hi-C or chromatin interaction analysis by paired-end tag sequencing (ChIA-PET). More-over, molecular networks have been successfully used to improve the inference accuracy of causal coding variants.^39–42^ However, although biologically intuitive, the complex interactions reflected by the underlying GRNs in which noncoding rSNPs take effect, namely, interactions among transcription factors and their target genes, are largely not taken into account in existing algorithms for functional SNP identification.^43^ We endeavored to capture such network-contextual information as new SNP-level features in CERENKOV3.

#### CERENKOV3:

CERENKOV3 takes advantage of newly engineered features reflecting SNP clustering and SNP network context and thereby improves rSNP prediction performance over our previous approaches, CERENKOV^17^ and CERENKOV2.^16^ As in our previous approaches, in CERENKOV3 we use regularized gradient boosted decision trees (XGBoost) as the base classifier due to its superior speed and performance. We combined our original 248-dimensional feature matrix with two new types of features derived from clustering and molecular networks, respectively: (1) “locus size”, a static feature based on the number of SNPs within a locus; and (2) a pair of dynamic features extracted by *node2vec*,^44^ an algorithm for learning continuous feature representations for nodes based on network random walks. We constructed a weighted molecular network using the the data sources 4DGenome,^45^ Encyclopedia of DNA Elements (ENCODE), and Genotype Tissue Expression (GTEx) for SNP-gene connections; and BioGRID,^46^ Coexpedia^47^ and HumanNet^48^ for gene-gene connections. We treated the edge weights as hyperparameters that we tuned in the method (see Methods).

## 2. Methods

### Reference SNP set and annotation features

A set of experimentally validated SNPs is fundamental to our supervised-learning method. In this work, we used the OSU18 SNP set that we first used in CERENKOV2^16^ and that is specifically designed to represent the computational task of post-GWAS rSNP identification. Loci are partitioned naturally by our filtering procedure when choosing SNPs: we only included SNPs within 50 kbp of an rSNP; this partitioning scheme also guarantees the possibility of locus sampling,^17^ a group-wise sampling technique that we implemented to assign SNPs to cross-validation (CV) folds by locus. After we analyzed the repeated locus-sampling based CV performance, we found that, whichever fold it was assigned, the locus with ID *chr5_30* (internally meaning the 30th locus on chromosome 5) would hinder the validation performance. From the OSU18 SNPs we pruned one locus (chr5_30) because it contained an anomalously high number of rSNPs (143) that lack supporting documentation in the source database (ClinVar). With that exclusion, the overall class balance of the remaining 38,795 OSU18 SNPs is *~*15.26 (ratio of control SNPs, or cSNPs, to rSNPs). As our baseline set of features, we obtained the 248 SNP annotation features from the CERENKOV2^16^ feature pipeline. For comparison purposes, we also extracted 175 SNP-level features from the GWAVA^19^ software.

### Clustering-derived feature: locus sizes

As mentioned above, when collecting the negative examples, i.e. the cSNPs, we only chose those that were in strong linkage disequilibrium (*r*^2^ ≥ 0.8) with, and located no more than 50 kbp distance from, an rSNP. This procedure provided us a natural way of clustering: we first sorted all the OSU18 SNPs by their locations per chromosome. Then, for any pair of neighboring SNPs on the same chromosome, if the distance between their chromosome positions is greater than 50 kbp, we divided them into two separate loci. In this way, we partitioned the OSU18 SNP set into 1290 loci, each containing approximately 30 SNPs on average. Then for each SNP, the number of SNPs within its locus is computed as the “locus size” feature.

### Construction of molecular networks

**Fig. 1.**
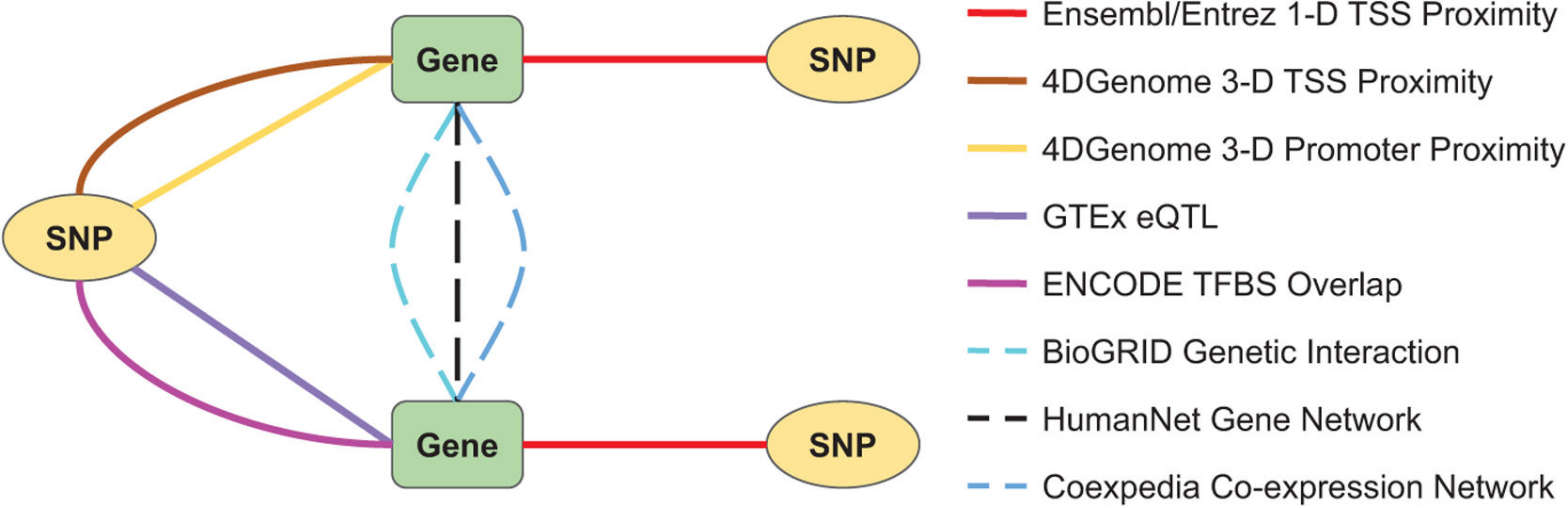
Data sources and types of relations used in to construct CERENKOV3 network.

We constructed an undirected SNP-proximal molecular network for CERENKOV3 as follows (Fig. 1): First, vertex types were limited to SNPs and genes in order to reduce constructional and computational overhead. Second, we integrated eight data sources of SNP-gene interactions (five interaction types using data from four sources, namely, 4DGenome,^45^ GTEx, Ensembl, and ENCODE) and gene-gene interactions (three sources) in order to maximize the connectedness of the network. Third, under the premise that different data sources are likely to have different degrees of relevance/informativeness for the rSNP prediction task, we assigned edges numerical weights according to the relation types they represented (see colored edges in Fig. 1); we treated these weights as hyperparamters of our classifer that we tuned empirically to maximize performance (see Sec. Machine learning pipeline and hyperparameter tuning). To construct the CERENKOV3 network, we used as vertices the pruned OSU18 SNP set and all human genes from Ensembl (release GRCh37). We mapped Ensembl gene IDs to NCBI IDs using BioMart as needed for integrating gene-gene interaction data sources.

### Detailed procedure for obtaining SNP-gene edges:

- For any single SNP *s*, if among all candidate gene vertices there is a gene *g* whose transcription start site (TSS) lies closest downstream to that gene, we drew an edge between *s* and *g*. We call this a “nearest-gene” SNP-gene relation, as it is based on SNP-TSS proximity.
- 4DGenome is a public database of chromatin interaction records that contains over three million human chromatin interactions curated from a comprehensive collection of 3C, 4C, 5C, ChIA-PET, Hi-C and IM-PET^49^ studies. If a SNP *s* and the TSS of a gene *g* exclusively located in two interacted regions reported by 4DGenome, we added a *s*–*g* connection in the network. Furthermore, for any gene *g*, we defined the promoter region to span the range from 2000 bp upstream to 500 bp downstream of its TSS. Similarly, for any pair of interacted regions reported by 4DGenome, if the genomic region contains a SNP *s* and partially overlaps with a gene *g*’s promoter section exclusively, such an *s*-*g* edge will also be included in our CERENKOV3 network. We call these two types of relations *4DGt* (for TSS proximity) and *4DGp* (for promoter proximity), respectively.
- GTEx is a comprehensive public resource to study tissue-specific gene expression and regulation. GTEx defines “eGenes” as genes with at least one SNP in *cis* significantly associated, at a false discovery rate (FDR) of *≤* 0.05, with expression differences of that gene. We used single-tissue *cis*-eQTL data from GTEx Analysis V7 and we incorporated all SNP-eGene associations into our CERENKOV3 network as edges.
- The last set of SNP-gene edges were obtained from connections through overlapping transcription factor binding sites (TFBS) using the UCSC Genome Browser and MyGene.info application programming interface (API). First, we used an inner join between the *All SNP* (build 146) and *Transcription Factor ChIP-seq Clusters V3* tables of the GRCh37 assembly from the UCSC Genome Browser to obtain all TFBS symbols overlapping with our pruned OSU18 SNP set; then we used MyGene.info API in order to find all genes that are translated into the corresponding transcription factors.

### Detailed procedure for obtaining gene-gene edges:

We directly obtained gene-gene edges from BioGRID,^46^ Coexpedia^47^ and HumanNet.^48^

- BioGRID is an online biological interaction repository with data compiled through comprehensive curation efforts. We used version 3.5.171 to extract all gene-gene pairs which participates in the interactions reported and augmented our network.
- Coexpedia and HumanNet (v2) are two gene co-expression databases and serve as a natural source of gene-gene edges.

### Network-derived features

Once the molecular network was constructed and a set of edge weight hyperparameters assigned (within the context of a hyperparameter search algorithm), we used *node2vec*^44^ to extract low-dimension continuous representations for each network vertex. Specifically, through a set of parameters controlling the usage of breadth-first and depth-first searches, *node2vec* provides a way of balancing the exploration-exploitation tradeoff when generating random walks for each vertex. Once the random walks are completed, *node2vec* calls *word2vec*,^50^ a word embedding algorithm, to generate embeddings on the string representations of the random walks. The dimension of *node2vec*’s output, i.e., the number of network-derived features, is not determined in advance; instead we optimized it within the hyperparameter search.

### Machine learning pipeline and hyperparameter tuning

**Fig. 2.**
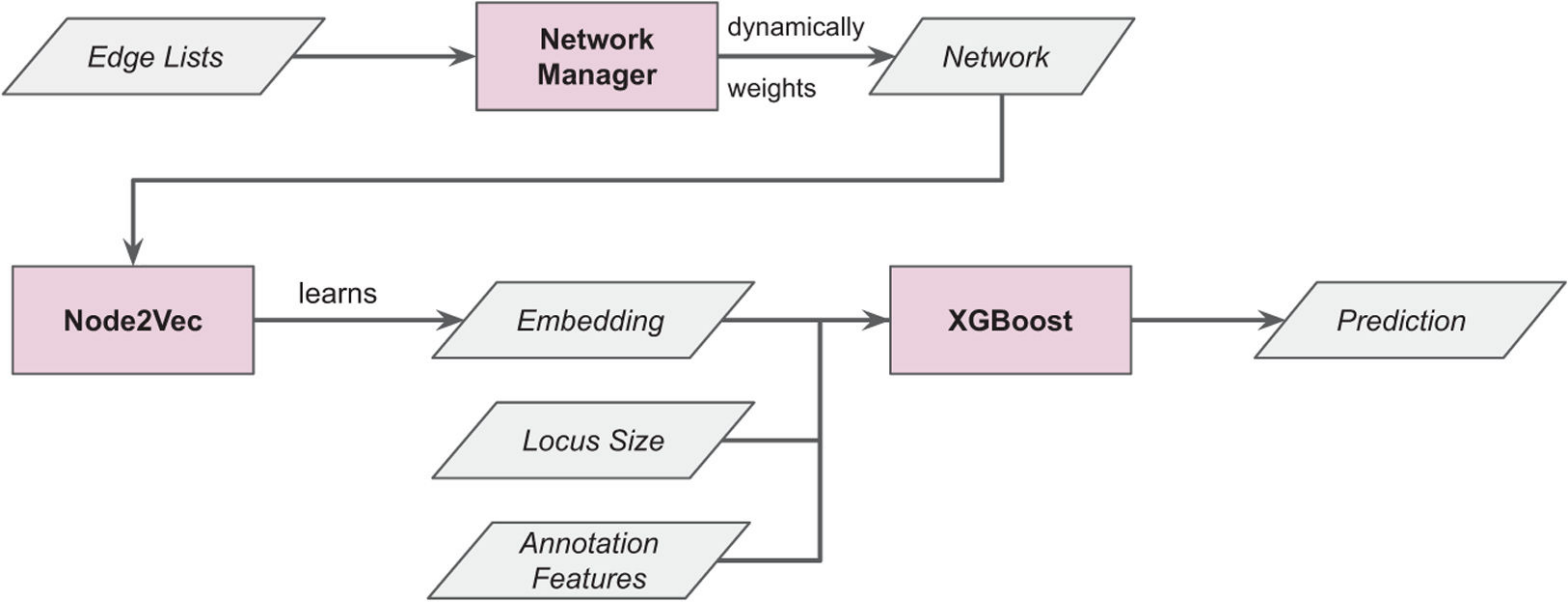
Pipeline of CERENKOV3 machine learning approach.

As shown in Fig. 2, the pipeline of CERENKOV3 includes three major steps:

1. The unweighted network is saved in edge-list format and then assigned weights dynamically according to the types of edges, i.e., the types of SNP-gene or gene-gene relations.
2. The weighted network is sent to *node2vec* and the embeddings are generated and output as new features for the classifier.
3. The network-derived features and clustering-derived feature, locus sizes, are integrated with the baseline 248-dimension SNP features. The combined feature matrix is input to the XGBoost classifier within the context of a replicated, locus sampling-based, five-fold cross-validation training process, with performance measures obtained on the validation sets.

The whole pipeline is wrapped into a custom scikit-learn estimator object, whose three sets of hyperparameters are as follows:

1. Edge weights, i.e., *w*_*NG*_ (for the *nearest-gene* relations), *w*_4*DGt*_, *w*_4*DGp*_ (for SNP-gene relations extracted from 4DGenome data), *w*_*GTEx*_, *w*_*TFBS*_ (for SNP-gene relations extracted through overlapping TFBS), *w*_*bg*_, *wcoexp* and *w*_*hn*_ (for gene-gene relations extracted from BioGRID, Coexpedia and HumanNet, respectively).
2. The hyperparameters of *node2vec*, including *d* (the number of output dimensions), *r* (the number of random walks for each vertex), *l* (the length of each random walk), *k* (the context window size when calling *word2vec*), *p* (the “return” degree, controlling the probability to go back to the visted vertex) and *q* (the “inout” degree, controlling the probability to explore undiscovered parts of the network).
3. The hyperparameters of XGBoost as below: max_depth (the maximum tree depth for base learners), learning_rate, n_estimators (the number of trees to fit), gamma (the minimum loss reduction required to make a further partition on a leaf node of the tree), subsample (subsample ratio of the training instance) and colsample_bytree (the subsample ratio of columns when constructing each tree).

Considering high dimensionality (20) of the hyperparameter space, we used a random search method^51^ to approximate the optimal configuration. The random search works on the assumption that 1% of the hyperparameter configuratoins will lead to close-to-optimal performance. Based on this assumption, with *n ≥* 240 trials, we would expect to find a close-to-optimal configuration with a high probability of 1 − (1 − 0.01)^*n*^ > 0.99.

For the CERENKOV3 machine learning pipeline, we used a combination of bash, bedtools (v2.25.0), the R statistical computing environment (version 3.4.4), scikit-learn (version 0.21.2) and Python 3.5.2, all under Ubuntu 16.04. In addition, for the purpose of comparison, we also generated features for the pruned OSU18 SNP set with the GWAVA^19^ program and then applied Random Forest algorithm with R package ranger version 0.6.0 with the published hyperparameters. To make a fair comparison, we adapted the same cross-valiation settings, fold assignments, and performance measurements for all classifiers.

## 3. Results

### Random search in hyperparameter space

We carried out a 240-trial random search on hyperparameters with XGBoost on a basis of ten-fold replicated, locus sampling-based, five-fold cross-validation and esetimated the optimal hyperparameters as shown below.

1. For edge weights, *w*_*NG*_ = 0.1, *w*_4*DGt*_ = 0.3, *w*_4*DGp*_ = 3.0, *w*_*GTEx*_ = 0.3, *w*_*TFBS*_ = 0.1, *w*_*bg*_ = 3.0, *wcoexp* = 0.3 and *w*_*hn*_ = 0.3.
2. For *node2vec*, *d* = 2, *r* = 12, *l* = 6, *k* = 4, *p* = 4 and *q* = 8.
3. For XGBoost, max_depth=10, learning_rate=0.1, n_estimators=100, gamma=10, subsample=1.0 and colsample_bytree=0.3.

For edge weights, we set the options for random search within a set of real numbers {0.0, 0.1, 0.3, 1.0, 3.0}. The optimized edge weights appear to emphasize snp-gene promoter-proximity edges through 4DGenome data and gene-gene edges from BioGRID. In terms of *node2vec* parameters, we found in general that lower dimensions of output (*d*) and longer distances of random walks (*l*) perform best. In addition, the optimal combination of *p* = 4 and *q* = 8 means probablistically in our constructed network, more breadth-first searches were carried out than depth-first ones in the optimal configuration.

### Analysis of newly engineered features

**Fig. 3.**
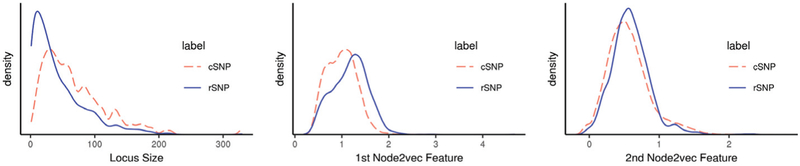
Kernel density estimated distributions of locus sizes and two network-derived features, for the two sets of ground-truth SNPs, rSNPs (solid line) and cSNPs (dashed line).

For each SNP, we obtained the locus size and the optimal two-dimensional embedding returned by *node2vec*. We first analyzed these three new features for the two SNP classes (rSNPs and cSNPs) using kernel density estimation for feature values conditioned on the class label (rSNP or cSNP) of the reference SNP. As seen in Fig. 3, there are evident likelihood differences (particularly reflecting differences in the skewness and kurtoses of the distributions) that could be exploited by XGBoost. For locus sizes, the feature distribution for rSNPs are slightly more left-shifted and more leptokurtic than the distributions for cSNPs; in terms of the first network-derived feature, the distribution for rSNPs is more shifted to the right; for the second network-derived feature, both of the distributions are more leptokurtic than those of the first features and similarly the distribution for rSNPs are more right-shifted.

### Comparison of performance

**Fig. 4.**
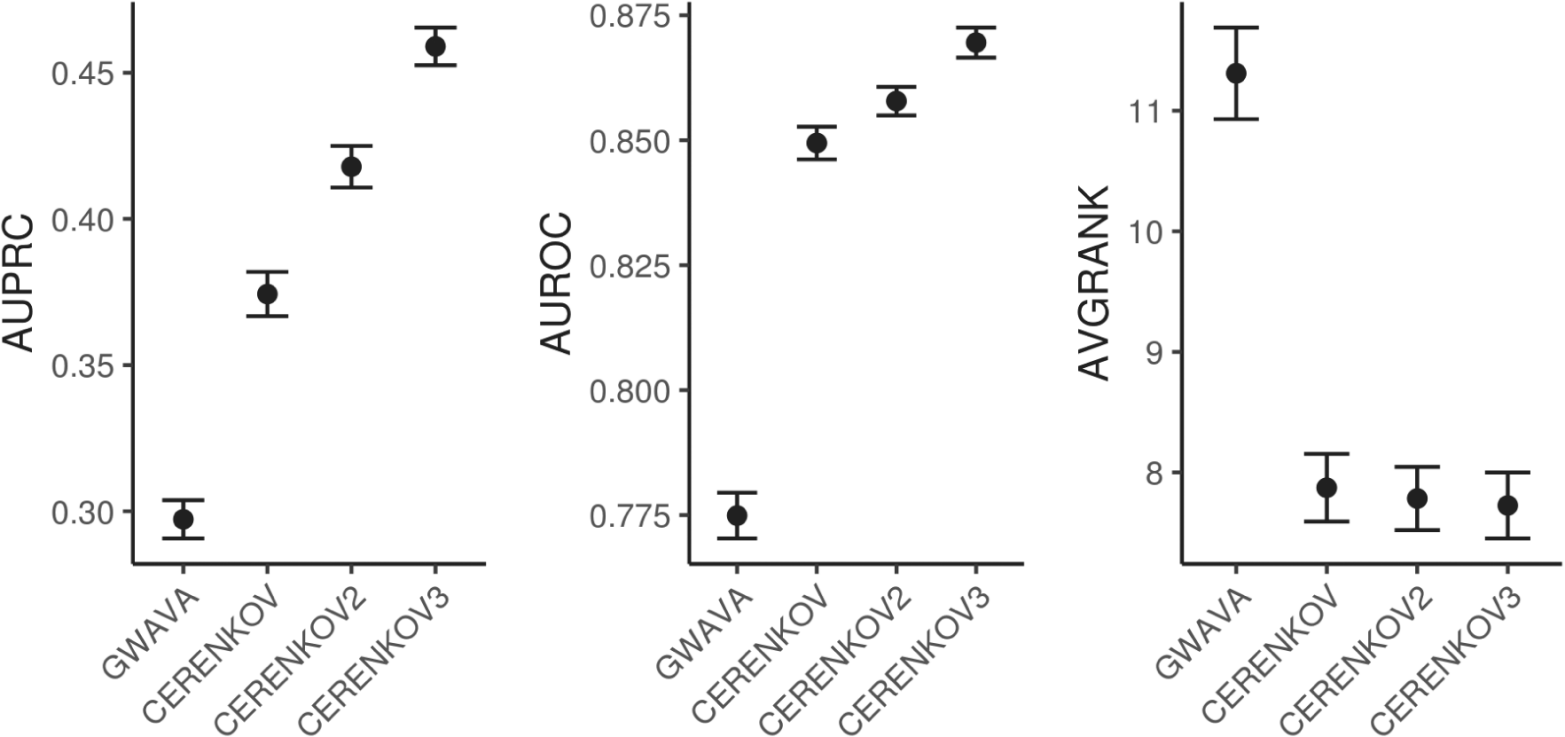
Performance of GWAVA, CERENKOV, CERENKOV2 and CERENKOV3 on the OSU18 reference SNP set, by three performance measures. Error bars denote *±* two standard errors from ten replications of five-fold CV.

Using the above-described optimal hyperparameters and cross-validation framework, we compared the performance of GWAVA, CERENKOV, CERENKOV2, and CERENKOV3 in terms of AUPVR, AUROC, and AVGRANK (Fig. 4). CERENKOV3 was the best-performing algorithm overall, significantly outperforming GWAVA (which was the best-performing of the nine competing algorithms in our previous study^17^). Comparing the performance of CERENKOV3 with CERENKOV2, we see that the inclusion of the three new features improved validation-set AUPRC from 0.418 to 0.459 (*p* < 10^−35^) and AUROC from 0.858 to 0.870 (*p* < 10^−19^); in terms of AVGRANK, although the improvement is not statistically significant (*p* = 0.30) when compared to that of CERENKOV2, AVGRANK decreased from CERENKOV’s 7.873 to a lower 7.726 in CERENKOV3 (lower is better for AVGRANK;^17^ *p <* 0.001). These results indicate that the addition of the three SNP clustering and molecular network-based features significantly improve rSNP recognition performance.

## 4. Conclusion and discussion

We have demonstrated, using side-by-side comparisons on identical assignments of SNPs to cross-validation folds, that CERENKOV3’s performance exceeds that of our previous CERENKOV, by both classical global rank-based measures (AUPRC and AUROC) and by the GWAS-oriented performance measure, AVGRANK. In particular, CERENKOV3’s validation-set AUPRC performance, 0.459, is a significant improvement over CERENKOV2’s AUPRC of 0.418 on the same pruned reference SNP set. These results reveal CERENKOV3’s ability, by virtue of its novel features based on clustering and molecular networks, to contribute to solving the problem of identifying candidate causal noncoding SNPs in GWAS summary regions.

We anticipate that CERENKOV3’s performance may be further improved through several possible enhancements. An appealing extension would be to combine deep neural network-based approaches based on the local 1 kbp sequence haplotype (recognizing that the local haplotype provides important correlates of functional SNP alleles^52^), with CERENKOV3’s current set of 251 SNP features. Our previous work^31^ has demonstrated that a classifier (Res2s2am) based on a deep residual network architecture has state-of-the-art performance on the related problem of discriminating trait-associated noncoding SNPs from control noncoding SNPs. Another direction of improvement is to continue feature engineering from clustering and networks. For example, currently, graph neural networks (GNN) are showing promise for integrating the SNP annotaion features and the connections between them. With GNN, it is possible to carry out representation learning on annotation features through graph embedding. The complete source code for CERENKOV3 is publicly available under an open-source license via GitHub at https://github.com/ramseylab/cerenkov3.

## References

[1] Welter D, MacArthur J, Morales J, Burdett T, Hall P, Junkins H, Klemm A, Flicek P, Manolio T, Hindorff L and Parkinson H, The NHGRI GWAS Catalog, a curated resource of SNP-trait associations, Nucleic Acids Res 42, D1001 (2014), accessed in 2016. 24316577 10.1093/nar/gkt1229 PMC3965119

[2] Schaub MA, Boyle AP, Kundaje A, Batzoglou S and Snyder M, Linking disease associations with regulatory information in the human genome, Genome Res 22, 1748 (2012). 22955986 10.1101/gr.136127.111 PMC3431491

[3] Maurano MT, Humbert R, Rynes E, Thurman RE, Haugen E, Wang H, Reynolds AP, Sandstrom R, Qu H, Brody J , Systematic localization of common disease-associated variation in regulatory DNA, Science 337, 1190 (2012). 22955828 10.1126/science.1222794 PMC3771521

[4] Stranger BE, Stahl EA and Raj T, Progress and promise of genome-wide association studies for human complex trait genetics, Genetics 187, 367 (2011). 21115973 10.1534/genetics.110.120907 PMC3030483

[5] Xu H, Gregory SG, Hauser ER, Stenger JE, Pericak-Vance MA, Vance JM, Züchner S and Hauser MA, SNPselector: a web tool for selecting SNPs for genetic association studies., Bioinformatics 21, 4181 (November 2005). 16179360 10.1093/bioinformatics/bti682 PMC1361283

[6] Macintyre G, Bailey J, Haviv I and Kowalczyk A, is-rSNP: a novel technique for *in silico* regulatory SNP detection, Bioinformatics 26, i524 (2010). 20823317 10.1093/bioinformatics/btq378 PMC2935445

[7] Xiao R and Scott LJ, Detection of cis-acting regulatory SNPs using allelic expression data., Genetic epidemiology 35, 515 (September 2011). 21769929 10.1002/gepi.20601 PMC4372992

[8] Riva A, Large-scale computational identification of regulatory SNPs with rSNP-MAPPER, BMC Genomics 13 Suppl 4, p. S7 (2012). 10.1186/1471-2164-13-S4-S7 PMC3303742 22759655

[9] Li MJ, Wang LY, Xia Z, Sham PC and Wang J, GWAS3D: Detecting human regulatory variants by integrative analysis of genome-wide associations, chromosome interactions and histone modifications, Nucleic Acids Res 41, W150 (2013). 23723249 10.1093/nar/gkt456 PMC3692118

[10] Bryzgalov LO, Antontseva EV, Matveeva MY, Shilov AG, Kashina EV, Mordvinov VA and Merkulova TI, Detection of regulatory SNPs in human genome using ChIP-seq ENCODE data, PLOS ONE 8, p. e78833 (2013). 24205329 10.1371/journal.pone.0078833 PMC3812152

[11] Kircher M, Witten DM, Jain P, O’Roak BJ, Cooper GM and Shendure J, A general framework for estimating the relative pathogenicity of human genetic variants, Nature Genet 46, 310 (2014). 24487276 10.1038/ng.2892 PMC3992975

[12] Gulko B, Hubisz MJ, Gronau I and Siepel A, A method for calculating probabilities of fitness consequences for point mutations across the human genome., Nature Genetics 47, 276 (2015). 25599402 10.1038/ng.3196 PMC4342276

[13] Lee D, Gorkin DU, Baker M, Strober BJ, Asoni AL, McCallion AS and Beer MA, A method to predict the impact of regulatory variants from DNA sequence, Nature Genet 47, 955 (2015), gkm-SVM. 26075791 10.1038/ng.3331 PMC4520745

[14] Quang D, Chen Y and Xie X, DANN: a deep learning approach for annotating the pathogenicity of genetic variants., Bioinformatics 31, 761 (2015). 25338716 10.1093/bioinformatics/btu703 PMC4341060

[15] Ionita-Laza I, McCallum K, Xu B and Buxbaum JD, A spectral approach integrating functional genomic annotations for coding and noncoding variants., Nature Genetics 48, 214 (2016). 26727659 10.1038/ng.3477 PMC4731313

[16] Yao Y, Liu Z, Wei Q and Ramsey SA, Cerenkov2: improved detection of functional noncoding snps using data-space geometric features, BMC bioinformatics 20, p. 63 (2019). 30727967 10.1186/s12859-019-2637-4 PMC6364436

[17] Yao Y, Liu Z, Singh S, Wei Q and Ramsey SA, Cerenkov: Computational elucidation of the regulatory noncoding variome, in Proceedings of the 8th ACM International Conference on Bioinformatics, Computational Biology,and Health Informatics, (ACM, Corvallis, OR, August 2017).

[18] Montgomery SB, Griffith OL, Schuetz JM, Brooks-Wilson A and Jones SJM, A survey of genomic properties for the detection of regulatory polymorphisms, PLOS Comput Biol 3, p. e106 (2007). 17559298 10.1371/journal.pcbi.0030106 PMC1892352

[19] Ritchie GRS, Dunham I, Zeggini E and Flicek P, Functional annotation of noncoding sequence variants, Nature Methods 11, 294 (2014). 24487584 10.1038/nmeth.2832 PMC5015703

[20] Peterson TA, Mort M, Cooper DN, Radivojac P, Kann MG and Mooney SD, Regulatory Single-Nucleotide Variant Predictor Increases Predictive Performance of Functional Regulatory Variants, Hum Mutat 37, 1137 (2016). 27406314 10.1002/humu.23049 PMC6192032

[21] Quang D and Xie X, DanQ: a hybrid convolutional and recurrent deep neural network for quantifying the function of DNA sequences, Nucleic Acids Res 44, p. e107 (2016). 27084946 10.1093/nar/gkw226 PMC4914104

[22] Krawczak M and Cooper DN, The human gene mutation database, Trends in Genetics 13, 121 (1997). 9066272 10.1016/s0168-9525(97)01068-8

[23] Montgomery SB, Griffith OL, Sleumer MC, Bergman CM, Bilenky M, Pleasance ED, Prychyna Y, Zhang X and Jones SJM, ORegAnno: an open access database and curation system for literature-derived promoters, transcription factor binding sites and regulatory variation, Bioinformatics 22, 637 (2006). 16397004 10.1093/bioinformatics/btk027

[24] Landrum MJ, Lee JM, Riley GR, Jang W, Rubinstein WS, Church DM and Maglott DR, ClinVar: public archive of relationships among sequence variation and human phenotype, Nucleic Acids Res 42, D980 (2014). 24234437 10.1093/nar/gkt1113 PMC3965032

[25] Andersen MC, Engström PG, Lithwick S, Arenillas D, Eriksson P, Lenhard B, Wasserman WW and Odeberg J, In silico detection of sequence variations modifying transcriptional regulation, PLOS Comput Biol 4, p. e5 (2008). 18208319 10.1371/journal.pcbi.0040005 PMC2211530

[26] Torkamani A and Schork NJ, Predicting functional regulatory polymorphisms, Bioinformatics 24, 1787 (2008). 18562267 10.1093/bioinformatics/btn311 PMC2732211

[27] Zhao Y, Clark WT, Mort M, Cooper DN, Radivojac P and Mooney SD, Prediction of functional regulatory SNPs in monogenic and complex disease, Hum Mutat 32, 1183 (2011). 21796725 10.1002/humu.21559 PMC3957483

[28] Battle A, Mostafavi S, Zhu X, Potash JB, Weissman MM, McCormick C, Haudenschild CD, Beckman KB, Shi J, Mei R, Urban AE, Montgomery SB, Levinson DF and Koller D, Characterizing the genetic basis of transcriptome diversity through RNA-sequencing of 922 individuals, Genome Res 24, 14 (2014). 24092820 10.1101/gr.155192.113 PMC3875855

[29] Zhou J and Troyanskaya OG, Predicting effects of noncoding variants with deep learning-based sequence model, Nature Methods 12, 931 (2015). 26301843 10.1038/nmeth.3547 PMC4768299

[30] Ryan NM, Morris SW, Porteous DJ, Taylor MS and Evans KL, SuRFing the genomics wave: an R package for prioritising SNPs by functionality, Genome Med 6, p. 79 (2014). 25400697 10.1186/s13073-014-0079-1 PMC4224693

[31] Liu Z, Yao Y, Benjamin W, Wei Q and Ramsey SA, Res2s2am: Deep residual network-based model for identifying functional noncoding snps in trait-associated regions, in Pacific Symposium on Biocomputing, 2019. 30864312

[32] Lee S-I, Dudley AM, Drubin D, Silver PA, Krogan NJ, Pe’er D and Koller D, Learning a Prior on Regulatory Potential from eQTL Data, PLOS Genet 5, p. e1000358 (2009). 19180192 10.1371/journal.pgen.1000358 PMC2627940

[33] Shin S and Keleş S, Annotation Regression for Genome-Wide Association Studies with an Application to Psychiatric Genomic Consortium Data., Statistics in biosciences 9, 50 (6 2017). 28781711 10.1007/s12561-016-9154-z PMC5542423

[34] Li MJ, Yan B, Sham PC and Wang J, Exploring the function of genetic variants in the non-coding genomic regions: approaches for identifying human regulatory variants affecting gene expression, Brief Bioinformatics 16, 393 (2015). 24916300 10.1093/bib/bbu018

[35] Nicolae DL, Gamazon E, Zhang W, Duan S, Dolan ME and Cox NJ, Trait-Associated SNPs Are More Likely to Be eQTLs: Annotation to Enhance Discovery from GWAS, PLOS Genet 6, p. e1000888 (2010). 20369019 10.1371/journal.pgen.1000888 PMC2848547

[36] Chen T and Guestrin C, XGBoost: A scalable tree boosting system, arXiv.org 1603.02754, 1 (2016).

[37] Buzdugan L, Kalisch M, Navarro A, Schunk D, Fehr E and Bühlmann P, Assessing statistical significance in multivariable genome wide association analysis, Bioinformatics 32, 1990 (2016). 27153677 10.1093/bioinformatics/btw128 PMC4920127

[38] Hess M, Lenz S, Blätte TJ, Bullinger L and Binder H, Partitioned learning of deep boltzmann machines for snp data, Bioinformatics 33, 3173 (2017). 28655145 10.1093/bioinformatics/btx408

[39] Lee I, Blom UM, Wang PI, Shim JE and Marcotte EM, Prioritizing candidate disease genes by network-based boosting of genome-wide association data, Genome research 21, 1109 (2011). 21536720 10.1101/gr.118992.110 PMC3129253

[40] Linghu B, Snitkin ES, Hu Z, Xia Y and DeLisi C, Genome-wide prioritization of disease genes and identification of disease-disease associations from an integrated human functional linkage network, Genome biology 10, p. R91 (2009). 19728866 10.1186/gb-2009-10-9-r91 PMC2768980

[41] Jia P, Zheng S, Long J, Zheng W and Zhao Z, dmgwas: dense module searching for genome-wide association studies in protein–protein interaction networks, Bioinformatics 27, 95 (2010). 21045073 10.1093/bioinformatics/btq615 PMC3008643

[42] Moreau Y and Tranchevent L-C, Computational tools for prioritizing candidate genes: boosting disease gene discovery, Nature Reviews Genetics 13, p. 523 (2012). 10.1038/nrg3253 22751426

[43] Gao L, Uzun Y, Gao P, He B, Ma X, Wang J, Han S and Tan K, Identifying noncoding risk variants using disease-relevant gene regulatory networks, Nature communications 9, p. 702 (2018). 10.1038/s41467-018-03133-y PMC5816022 29453388

[44] Grover A and Leskovec J, node2vec: Scalable feature learning for networks, in Proceedings of the 22nd ACM SIGKDD international conference on Knowledge discovery and data mining, 2016. 10.1145/2939672.2939754 PMC5108654 27853626

[45] Teng L, He B, Wang J and Tan K, 4dgenome: a comprehensive database of chromatin interactions, Bioinformatics 31, 2560 (2015). 25788621 10.1093/bioinformatics/btv158 PMC4514924

[46] Stark C, Breitkreutz B-J, Reguly T, Boucher L, Breitkreutz A and Tyers M, Biogrid: a general repository for interaction datasets, Nucleic acids research 34, D535 (2006). 16381927 10.1093/nar/gkj109 PMC1347471

[47] Yang S, Kim CY, Hwang S, Kim E, Kim H, Shim H and Lee I, Coexpedia: exploring biomedical hypotheses via co-expressions associated with medical subject headings (mesh), Nucleic acids research 45, D389 (2016). 27679477 10.1093/nar/gkw868 PMC5210615

[48] Hwang S, Kim CY, Yang S, Kim E, Hart T, Marcotte EM and Lee I, Humannet v2: human gene networks for disease research, Nucleic acids research 47, D573 (2018). 10.1093/nar/gky1126 PMC6323914 30418591

[49] He B, Chen C, Teng L and Tan K, Global view of enhancer–promoter interactome in human cells, Proceedings of the National Academy of Sciences 111, E2191 (2014). 10.1073/pnas.1320308111 PMC4040567 24821768

[50] Goldberg Y and Levy O, word2vec explained: deriving mikolov et al.’s negative-sampling word-embedding method, arXiv preprint arXiv:1402.3722 (2014).

[51] Bergstra J and Bengio Y, Random search for hyper-parameter optimization, Journal of Machine Learning Research 13, 281 (2012).

[52] Ward LD and Kellis M, Interpreting noncoding genetic variation in complex traits and human disease, Nature Biotechnol 30, 1095 (2012). 23138309 10.1038/nbt.2422 PMC3703467

